# Efficient 5′-3′ DNA end resection by HerA and NurA is essential for cell viability in the crenarchaeon *Sulfolobus islandicus*

**DOI:** 10.1186/s12867-015-0030-z

**Published:** 2015-02-14

**Authors:** Qihong Huang, Linlin Liu, Junfeng Liu, Jinfeng Ni, Qunxin She, Yulong Shen

**Affiliations:** State Key Laboratory of Microbial Technology, Shandong University, 27 Shanda Nan Rd., Jinan, 250100 P. R. China; Archaea Centre, Department of Biology, University of Copenhagen, Ole MaaløesVej 5, Copenhagen N, DK-2200 Denmark

**Keywords:** Homologous recombination repair, ATPase, Helicase, Nuclease, HerA, NurA, Archaea

## Abstract

**Background:**

ATPase/Helicases and nucleases play important roles in homologous recombination repair (HRR). Many of the mechanistic details relating to these enzymes and their function in this fundamental and complicated DNA repair process remain poorly understood in archaea. Here we employed *Sulfolobus islandicus,* a hyperthermophilic archaeon, as a model to investigate the *in vivo* functions of the ATPase/helicase HerA, the nuclease NurA, and their associated proteins Mre11 and Rad50.

**Results:**

We revealed that each of the four genes in the same operon, *mre11*, *rad50*, *herA*, and *nurA*, are essential for cell viability by a mutant propagation assay. A genetic complementation assay with mutant proteins was combined with biochemical characterization demonstrating that the ATPase activity of HerA, the interaction between HerA and NurA, and the efficient 5′-3′ DNA end resection activity of the HerA-NurA complex are essential for cell viability. NurA and two other putative HRR proteins: a PIN (PilT N-terminal)-domain containing ATPase and the Holliday junction resolvase Hjc, were co-purified with a chromosomally encoded N-His-HerA *in vivo*. The interactions of HerA with the ATPase and Hjc were further confirmed by *in vitro* pull down.

**Conclusion:**

Efficient 5′-3′ DNA end resection activity of the HerA-NurA complex contributes to necessity of HerA and NurA in *Sulfolobus,* which is crucial to yield a 3′-overhang in HRR. HerA may have additional binding partners in cells besides NurA.

**Electronic supplementary material:**

The online version of this article (doi:10.1186/s12867-015-0030-z) contains supplementary material, which is available to authorized users.

## Background

Of the various types of DNA lesions, double-strand breaks (DSBs) are one of the most detrimental, capable of causing chromosomal rearrangements and eventually cell death if not repaired appropriately [1]. In eukaryotes, two major DSB repair pathways are known: non-homologous end joining (NHEJ) and homologous recombination (HR). The former is an error-prone process, while the latter mechanism exhibits high fidelity [2]. It has been suggested that in eukaryotes, DSBs that occur in the G1 phase of the cell cycle are most likely to be repaired via NHEJ, while those occurring in the S/G2 phase are preferentially processed via HRR [2,3].

HRR has been investigated extensively in bacteria and eukaryotes. Bacteria encode multiple pathways for DSB repair, including RecBCD, the primary HRR pathway, SbcC-SbcD, and one backup system, RecFOR [4,5]. The HRR pathway can be divided into five general steps: (1) recognition of the break sites and formation of a repair center (RC), (2) end-processing of the broken ends, (3) loading of RecA onto single-strand DNA, homology search, and strand invasion, (4) branch migration and resolution, or dissolution of the recombination intermediates and replication restart, and (5) disassembly of the recombination apparatus and segregation of sister chromosomes [5]. The eukaryotic HRR machinery is comprised of a core protein complex containing Mre11-Rad50-Xrs2/Mre11-Rad50-NBS1(MRX/N); the nucleases/helicases exodeoxyribonuclease 1 (Exo1/EXO1), Dna2/DNA2, and Sgs1/BLM; the recombinase Rad51/RAD51; and several other accessory and regulatory proteins [6]. The HRR pathway in eukaryotes proceed similarly with several steps of the bacterial pathway, but multiple layers of regulation exist to ensure these repair pathways are accurate and restricted to the appropriate cellular contexts [3]. In eukaryotes, DSBs are recognized by the MRX(N) complex which is involved in most DNA end-associated processes including damage checkpoint signaling, HR, NHEJ, telomere maintenance, and meiotic recombination [1]. DNA end processing is initiated by MRX/MRN in conjunction with Sae2/CtIP and proceeds along one of the two distinct pathways, Exo1/EXO1 or Dna2/DNA2-Sgs1/BLM, forming a long 3′-tail of single-stranded DNA (ssDNA) that is then utilized in Rad51-dependent strand exchange in HR [7-11].

Archaea appear to primarily encode the HRR pathway for DSB repair, since homologs of several eukaryotic HRR components have been identified, including Mre11 and Rad50 of the MR complex and the recombinase RadA [12,13], and homologs of the NHEJ proteins Ku70/80 are only present in a limited number of archaeal genomes [14,15]. The RecQ-like helicase Hjm (**H**olliday **j**unction **m**igration) and the 5′-flap endonuclease which has both endonuclease and 5′-3′ exonuclease activities have been identified in archaea [16,17]; however, it is unclear whether they are involved in dsDNA end resection. Intriguingly, two archaeal genes, *herA* and *nurA,* are implicated in HRR by their genetic association with *mre11* and *rad50* in thermophilic archaea [18]. This has been supported by biochemical characterization of the encoded proteins: HerA exhibits ATPase activity and some exhibit dipolar helicase activities, while NurA is a 5′-3′ ssDNA/double-stranded (ds) DNA exonuclease and ssDNA endonuclease [18-22]. Several studies have demonstrated that Mre11, Rad50, HerA, and NurA are capable of working in concert to process dsDNA *in vitro* [22-26]. Thus, HerA and NurA are regarded as the functional homologs of the eukaryotic Exo1/EXO1, Dna2/DNA2, and Sgs1/BLM proteins, and the archaeal Mre11-Rad50-HerA-NurA system can serve as a simple model system for studying HRR.

The functional role of these putative HRR proteins in archaea has been investigated only in a few reports [27-31]. However, gene function is still hypothesized based on the negative results of genetic analyses, such as the inability to isolate null mutants for *radA*, *mre11*, *rad50*, *herA*, and *nurA* in *Thermococcus kodakaraensis* or *radA, herA*, and *hjm* in *Sulfolobus islandicus* [29,32,33]. This is due in large part to the fact that suitable tools for conducting sophisticated analyses of gene function in archaea are still lacking. Recently, we reported a genetic complementation test for *S. islandicus* based on simvastatin selection [34]. This method can be utilized to analyze protein function by rescuing an essential gene deletion with expression of a mutant derivative from plasmids.

In this study, we analyzed the necessity of four genes putatively involved in DSB repair in *S. islandicus* REY15A, a genetic model for which the genome has been sequenced [35] and versatile genetic tools have been developed [36]. We revealed that all the four genes, *mre11*, *rad50*, *herA*, and *nurA* are essential for cell viability. Furthermore, we demonstrated that the ATPase activity of HerA, the interaction between HerA and NurA, and the high 5′-3′ exonuclease activities of the HerA-NurA complex are essential for cell viability. We provide further evidence that HerA and NurA form a complex *in vivo*. The co-purification of HerA with a PIN (PilT N-terminal)-domain containing ATPase and the Holliday junction resolvase Hjc implies that HerA may also be involved in the HJ processing.

## Methods

### Strains and growth conditions

*Sulfolobus islandicus* strain REY15A(E233S) (△*pyrEF* △*lacS*) (hereafter E233S) (Additional file 1: Table S1) [37] was grown at 75°C in STVyU medium containing mineral salts, 0.2% (wt/vol) sucrose (S), 0.2% (wt/vol) tryptone (T), a mixed vitamin solution, 0.005% (wt/vol) yeast extract (y) and 0.01% (wt/vol) uracil (U), as described previously [37]. SCVy medium where tryptone was replaced by casamino acid (C) was used for cultivating uracil prototrophic transformants. ATVy medium where sucrose was replaced with 0.2% (wt/vol) arabinose (A) was used for protein expression (Additional file 1: Table S1). The STVyU medium supplemented with 5-fluorotic acid (5-FOA) (STVyUF) was used for counter-selection of the *pyrEF* auxotroph. Phytagel (0.7% [wt/vol]) was used for making the culture plates. The strains carrying the simvastatin-resistant selection marker (Additional file 1: Table S1) were grown in a medium supplemented with 12 μM simvastatin (Hangzhou Deli Chemical, Hangzhou, China) as described previously [34].

### Construction of knockout plasmids

All plasmids used in this study are listed in Additional file 2: Table S2. The plasmids for gene knockout, pMID-*mre11*, pMID-*rad50*, and pMID-*nurA*, were constructed in the similar way as for pMID-*herA* [34]. All of the plasmids contained three homologous DNA arms: the integration (IN) arm, looping-out (OUT) arm, and target gene (TG) arm. The fragments were amplified using *S. islandicus* genomic DNA and their corresponding primers (Additional file 3: Table S3). After digestion with the corresponding restriction enzymes, these fragments were cloned into the knockout vector pMID which contains *pyrEF-lacS* selection markers [38].

### Construction of plasmids for protein overexpression in *Escherichia coli* and for the genetic complementation assay

The construction of plasmids for protein expression and for the genetic complementation assay are described in Additional file 4: Supplementary methods. Briefly, the vector pET29a and pSSR carrying a simvastatin-resistant marker *hmg* [34] were used as the vectors.

### Construction of a plasmid for the addition of a His-tag-coding sequence to 5′ end of chromosomal *herA* (*in situ* His-tagged)

The method for the construction of the plasmid pMIDHis-*herA* for HerA *in situ* His-tagging used the pUC19 as the original vector similar to that for the knockout plasmids. The plasmids contained three arms: two copies of L-arm (a 213 bp fragment at the upstream of the *herA* start codon), L-arm-1 and L-arm-2, and G-arm (a fragment of 5′ *herA* gene). The sequence of L-arm-1 was exactly the same as L-arm-2. L-arm-1 was cloned into pUC19 at the restriction sites of *Sal*I and *Mlu*I yielding pL-arm-1. The *herA* gene with N-terminal histidine coding sequence was amplified from pSeSDA-N-His-HerA (Additional file 2: Table S2), an expression plasmid carrying *herA* inserted at the restriction sites of *Cla*I and *Sal*I in pSeSD [39], with the primers 5′-6His-HerA-G-arm F/5′HerA-G-arm-*Sph*I R. L-arm-2, amplified with the primers 5′HerA L-arm-1-*Nco*I F/5′HerA L-arm-2-6His R, was ligated to the G-arm by SOE PCR. The fragment containing L-arm-2 and G-arm was cloned into pL-arm-1 at the *Nco*I and *Sph*I sites yielding the plasmid pMIDHis-*herA*.

### Transformation of *S. islandicus* strains

The expression plasmids or linearized knockout plasmids were transformed into *S. islandicus* cells by electroporation as previously described [37].

### X-gal assay

To detect the presence of the *lacS* marker in cells, X-Gal (5-bromo-4-chloro-3-indolyl-β-D-galactopyranoside) staining was performed as previously described [34].

### Mutant propagation assay

The gene necessity was determined by a mutant propagation assay as described with minor modifications [38]. Briefly, 5-FOA was added into the culture of the purified pMID transformant (pMID-T) at OD_600_ ~ 0.4 and the culture continued for counter-selection, resulting in enrichment culture 1 (En1). Cells of En1 were diluted with the same fresh media to OD_600_ ~ 0.1 after they grew to the stationary phase. The OD_600_ values were measured at indicated times until the culture grew to the stationary phase or died.

### Complementation assay of HerA and NurA mutants

pSSR vectors harboring genes encoding wild type HerA, NurA, or the site-directed mutants were used to complement the chromosomal *herA* or *nurA* deletion. The pSSR vector was transformed into pMID-*herA*-T or pMID-*nurA*-T and the transformants were selected on STVy plates supplemented with 12 μM simvastatin (STVy + sim). Single colonies were subsequently transferred into a 25 ml tube containing 10 ml of STVy + sim medium. After the culture grew, the cells were spread onto STVyUF + sim plates for counter selection. The colonies were picked and individually cultured in a tube containing 10 ml of STVyUF + sim medium and subsequently inoculated in a flask containing 50 ml of the same medium.

### Genotype verification of the complementation strains by PCR and sequencing

*Sulfolobus* genomic DNA of the complementation strain was extracted from 3 ml of culture using Bacterial DNA Kit from Omega Bio-Tek (Norcross, GA, USA). The genotype at the loci was determined by PCR with the locus flanking primers.

For verification by sequencing, plasmids were isolated from 20–30 ml of the *Sulfolobus* cell cultures with Plasmid Mini Kit I (Norcross, GA, USA). The plasmids were re-transformed to *E. coli* DH5α for amplification. More than three *E. coli* single colonies were picked for plasmid extraction. The target genes in the plasmids were sequenced by BGI (Shenzhen, China).

### Protein purification

The pET29a plasmids carrying *herA*, *nurA*, or their mutant genes were transformed into *E. coli* BL21 (DE3)-CodonPlus-RIL for expression. The procedures for induction and purification of the proteins from *E. coli* cells were described in the Additional file 4: Supplementary methods.

To purify HerA from an *in situ* N-His-tagged HerA E233S strain, 9 L of the cells cultivated in the STV medium were collected by centrifugation. The cells were disrupted by sonication and the soluble fraction was precipitated by ammonium sulfate (0.6 g/ml). The precipitate was re-suspended in buffer A (50 mM Tris-HCl pH 8.0, 100 mM NaCl) and purified by Ni-NTA column described in the Additional file 4: Supplementary methods. The eluted protein was pooled, concentrated and diluted in buffer A. The subsequent sample was loaded onto a Superdex™ 200 10/300 GL column which was pre-equilibrated with buffer A. The proteins fractions were analyzed by SDS-PAGE and Western blot.

### Analysis of HerA-NurA interaction by gel filtration chromatography

Physical interactions between HerA and NurA (wild type and I295L, I295E, F300Y, and F300E mutants) were detected by gel filtration. HerA (500 μg) and NurA (500 μg) were mixed and incubated at 60°C for 20 min in a total 500 μl of gel filtration buffer (20 mM Tris pH 8.0, 300 mM NaCl, 5% glycerol, 1 mM DTT). The mixtures were then spun down to remove any precipitated protein and loaded onto a Superdex™ 200 10/300 GL column pre-equilibrated by gel filtration buffer. The fractions (0.5 ml each) were collected and analyzed by 15% SDS-PAGE.

### DNA substrates for the activity assays

Three oligonucleotides were synthesized for preparation of substrates for the nuclease assays (Additional file 3: Table S3). Strand E (34-mer in length) was 5′ end labeled with [γ-^32^P-ATP] as previously described [22]. The labeled oligonucleotides were purified with Illustra™ Microspin™ G-25 columns (GE Healthcare, UK). The annealing was performed as previously described [22]. Various substrates were constructed by combinations of different oligonucleotides (Additional file 5: Table S4). The DNA substrates were stored at 4°C.

### ATPase activity assay

The ATPase activity assay was performed as previously described. SsDNA, dsDNA, or NurA was added to a final concentration of 20 nM in the reaction [22].

### The nuclease assay of HerA-NurA

The nuclease assay was performed in 20 μl reaction mixtures consisting of indicated amounts of wild-type HerA (or its mutants) and wild-type NurA (or its mutant D58A), 50 mM Tris-HCl, pH 8.0, 50 mM NaCl, 10 mM MgCl_2_, 1 mM DTT, 1 mM ATP, 0.001% BSA, and 1 nM [γ-^32^P]-labeled dsDNA. The mixture was incubated at 65°C for 30 min and then stopped by addition of a 5× loading buffer (50 mM EDTA, 0.5% SDS, 25% glycerol, and 0.025% bromophenol blue). The products were separated by electrophoresis in a native polyacrylamide gel at 120 V for 90 min in 1 × TBE. To examine the degradation products of the labeled ssDNA, the sample was analyzed by a 15% denatured polyacrylamide gel as previously described [21]. The electrophoresis was run at 300 V for 90 min in 1 × TBE. The gels were exposed to a phosphorimager and scanned with Typhoon 9410.

### Western blot analysis

Aliquots (50 μl for each) of the gel filtration fractions were mixed with 5 × SDS-PAGE loading buffer and loaded onto the gel for SDS-PAGE analysis. The proteins in the PAGE gel were transferred onto a PDVF membrane at 30 mA for 15 hrs at 4°C. The membrane was washed and incubated with a primary antibody and a secondary antibody anti-rabbit HRP-conjugate (HuaAn Biotechnology limited company, Hangzhou, China) following the standard protocol for Western blot. The protein-specific rabbit polyclonal antibodies were made by HuaAn Biotechnology limited company with a 15 aa synthetic peptide specific to each target protein as immunogens (Additional file 6: Table S5). The band was visualized with Immobilon™ Western Chemiluminescent HRP Substrate (Millipore Corporation, Billerica, MA, USA) and the images were obtained by Imagequant™ 400 (GE Healthcare, UK).

For assessment of HerA and NurA levels in cells by Western blot, cells from 0.5 ml E233S culture (OD_600_ ~ 0.4) were collected and lysed by mixing with 5 × SDS-PAGE loading buffer and boiled for 10 min. Total proteins were separated by SDS-PAGE for Western blot analysis as described above. Purified proteins were loaded as standards for quantification.

## Results

### *herA, mre11, rad50,* and *nurA* are all essential for cell viability

**Figure 1:**
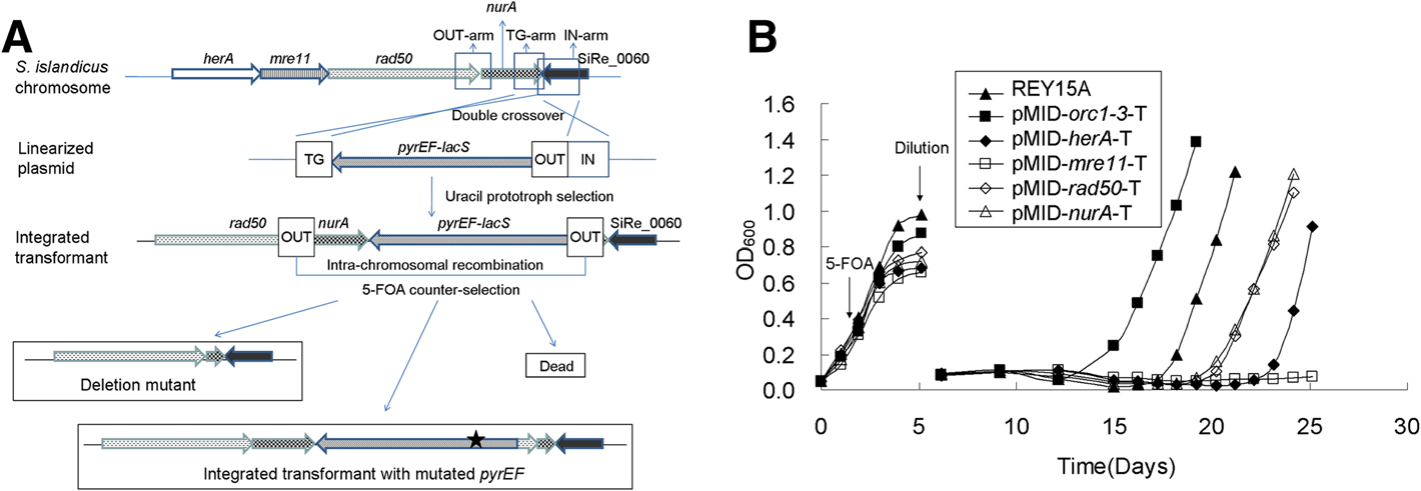
**The four HRR genes,** ***herA*** **,** ***mre11*** **,** ***rad50*** **, and** ***nurA*** **, which are encoded in a single operon, are all essential for cell viability. (A)** Schematic diagram depicting the construction of the strain used in the gene knockout analysis. Marker insertion and target gene deletion (MID) and a subsequent mutant propagation assay were utilized. The star indicates a putative spontaneous mutation site in *pyrEF*. **(B)** Mutant propagation assay for *pyrEF-lacS*-integrated transformants (pMID-*herA*-T, pMID-*mre11*-T, pMID-*rad50*-T, and pMID-*nurA*-T). *orc1-3*, a nonessential gene coding for one of the three replication initiation proteins, was used as a positive control, whereas REY15A is a reference for *pyrEF* mutation. Strains were cultivated in STVy medium supplemented with 2 mg/ml uracil. The counter selection chemical, 5-FOA, was added when OD_600_ reached 0.4. The culture was diluted with fresh medium to OD_600_ ~ 0.1 when the cells had grown to early stationary phase. Absorbance of the culture was measured.

It has been reported that each of the four genes in the *mre11* operon, namely *herA*, *mre11*, *rad50*, and *nurA* are possibly essential since deletion mutants were not obtainable (reviewed in Zhang *et al.* [33]). In these experiments, gene knockouts were tested by construction of a target gene-specific strain pMID-T and two-step selections (Figure 1A). A vector pMID (**m**arker **i**nsertion and target gene **d**eletion) containing a marker cassette (*pyrEF-lacS*) and three homologous arms (TG-arm, IN-arm and OUT-arm) specific to the target gene was constructed and a marker-integrated strain pMID-T was obtained by selection of a uracil prototroph (Figure 1A). The resulting strain pMID-T was subjected to the second selection of a uracil auxotroph by the addition of 5-FOA and uracil into the culture medium. If the targeted gene is successfully knocked out, cells are able to grow up easily under the second selection (Figure 1A). However, no deletion mutant for any of *herA, mre11, rad50,* or *nurA* was obtained after 5-FOA and uracil (Figure 1A).

To affirm the essentiality of the HRR genes, these recombinant pMID-T strains were employed in a mutant propagation assay [38]. If a gene is essential, no deletion mutant can be obtained, but spontaneous mutation at *pyrEF* would occur at a low frequency close to the corresponding rates reported for the protein-encoding genes of *E. coli* [40]. The mutant may then accumulate and the culture will grow, albeit at a slow rate (Figure 1A, bottom scheme). The culture will stop growing when the original growth rate is too low for the spontaneous mutants to accumulate (Figure 1A).

In the current study, two reference strains were used in the mutant propagation assay: the wild-type strain, REY15A [41], was used to investigate the growth of spontaneous *pyrEF* mutants, whereas pMID-*orc1-3*-T was used to investigate the growth of mutant cells of a nonessential target gene deletion [42] (Additional file 1: Table S1). It has been shown that *orc1-3*, which encodes a replication initiator protein, can be deleted in *S. islandicus* [42]. As shown in Figure 1B, pMID-*orc1-3*-T culture grew most rapidly, followed by the wild-type strain REY15A. Cultures of pMID-*rad50*-T, pMID-*nurA*-T, and pMID-*mre11*-T grew much slower than REY15A or not at all (Figure 1B). As for the wild-type strain REY15A, the growth of these pMID transformants was not due to deletion of the respective gene, but due to propagation of cells with a mutation in *pyrEF* (Figure 1A). The strain pMID-*mre11*-T never grew. As shown in Figure 1B, up to day 5, the enrichment of pMID-*mre11*-T caused the cells to grow more slowly than the others. We also noticed that colonies of this strain appeared later than others on plates. The integration of the marker cassette and OUT arm at the upstream of *mre11* locus may have affected its gene expression, resulting in growth retardation. The results demonstrate that all four putative HRR genes are essential for the viability of *S. islandicus*.

### ATPase activity is essential but not sufficient for the *in vivo* function of HerA

Recently, we developed a genetic complementation method for *S. islandicus* in which an essential gene deficiency in the chromosome is able to be rescued by ectopic expression of the wild-type protein from a plasmid [34]. In this method, a pMID-T strain (pMID-*herA-*T) of the target gene *herA* as described above was transformed with a pSSR plasmid (harboring a simvastatin resistance marker and expressing the wild-type gene) under selection by simvastatin. Upon selection of the transformed strain with uracil, 5-FOA, and simvastatin, the essential chromosomal gene together with the *pyrEF-lacS* maker was looped out by intra-chromosomal recombination, and the plasmid was maintained in the absence of antibiotics. Here we employed that system to test the possibility of rescuing *herA* deficiency with different HerA mutant proteins, and investigated the functions of the residues critical for various properties, including ATPase activity of HerA, nuclease activity of the HerA-NurA complex, and the interaction between HerA and NurA.

**Figure 2:**
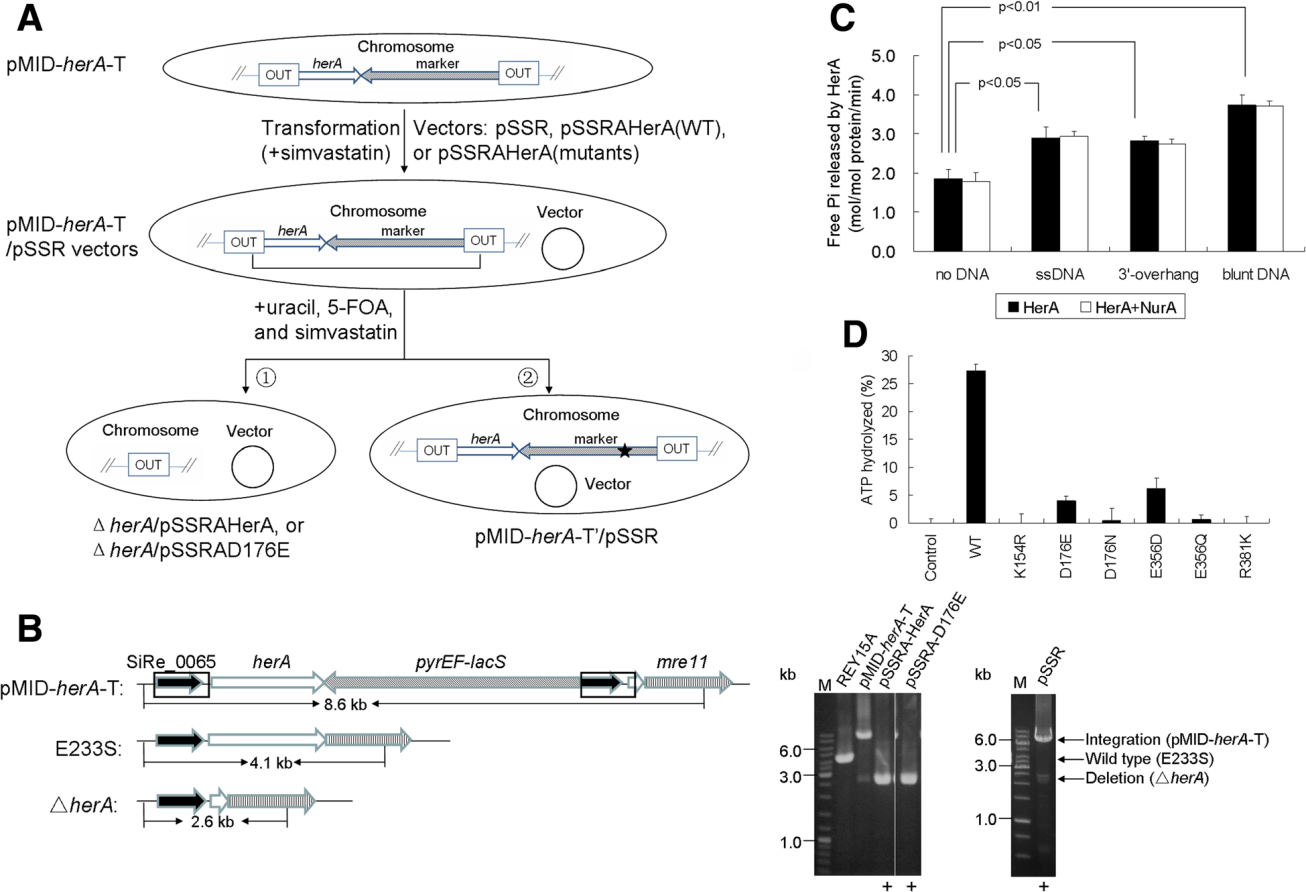
**A HerA(D176E) mutant with reduced ATPase activity is able to complement deletion of the wild-type** ***herA*** **gene. (A)** A schematic map depicting the procedures of the complementation analysis. pMID-*herA*-T cells were transformed with pSSR vectors. The cells were selected in the presence of simvastatin. Counter-selection was performed with solid medium containing 5-FOA, uracil, and simvastatin. Two genotypes of the cell grown up were indicated as ➀ and ➁. The star indicates a putative spontaneous mutation in *pyrEF*. **(B)** Genotype analysis of cells by PCR using *herA* flanking primers. The genomic DNA of each strain was isolated and used as the template. The pSSR plasmids in cells were isolated and amplified in *E. coli* DH5α for sequencing. Left panel, a schematic map showing the lengths of PCR products of different strains. Right panel, agarose gel electrophoresis of the PCR products. “+”, the designed plasmid maintained. **(C)** ATPase activity of HerA alone or with NurA in the absence or presence of DNA. The assay was performed as described in the Methods. Reaction products were separated using thin layer chromatography and analyzed by Phosphorimager. The means ± standard errors of three independent experiments and p values (n = 3) are shown. **(D)** ATPase activity of wild-type and mutant HerA proteins. The means ± standard deviations of three independent experiments are shown.

*In vitro* site-directed mutagenesis of archaeal HerA proteins has revealed conserved amino acid residues critical for its ATPase and helicase activities [18,22,23]. These amino acid residues are K154, D176, E356, and R381 of *S. islandicus* HerA, among which K154 and E356 are located in the Walker A and Walker B motifs, respectively. Point mutations at the four conserved sites were introduced by site-directed DNA mutagenesis and mutant genes encoding K154R, D176E, D176N, E356D, E356Q, or R381K substitutions were cloned into pSSR to create genetic complementation plasmids (Additional file 2: Table S2). The genetic host used for the experiment was pMID-*herA*-T, the recombinant strain carrying a marker-target gene cassette (*pyrEF-lacS-herA*) at the locus of *herA* gene (Figure 2A). A complementation plasmid was introduced into the host via transformation, and transformants were obtained by simvastatin selection.

Strikingly, only the transformants harboring pSSR, pSSRA-HerA or pSSRA-D176E were obtained, while no transformants with pSSR carrying any of other five mutants grew successfully after transformation. We have shown that ectopic wild type HerA was expressed in higher amounts in the cell than chromosomal HerA [34]. It is likely that the mutant protein was also in much higher abundance than the chromosome-coded HerA in the cells if the cells were transformed with the plasmid and the gene expression was induced. Loss of the ATPase and helicase activity in the HerA mutant was highly lethal to the cells. In a recent report, an *in vitro* mutant-doping assay showed that increasing the ratio of *S. solfataricu*s K154A to WT HerA resulted in an exponential drop in the proportion of unwound DNA substrate, where the ratio of 1:1 almost inactivated the unwinding activity [43]. The result is consistent with our *in vivo* data. Our results are also in good agreement with the above result that *herA* is essential for cell viability.

The three transformants above were then subjected to counter-selection against *pyrEF* on plates supplemented with uracil, 5-FOA, and simvastatin (Figure 2A). As shown in Figure 2B, two genotypes survived the selection. One genotype represents a Δ*herA* carrying plasmid harboring pSSRA-HerA or pSSRA-D176E and the other represents pMID-*herA*-T cells carrying a *pyrEF* mutation with the empty vector pSSR (Figure 2A). PCR and sequencing confirmed that the culture with pSSRA-HerA(D176E) contained a deleted chromosomal *herA* allele as that of pSSRA-HerA, and the plasmid carried the D176E *herA* substitution (Figure 2B). Thus, these results demonstrate that only wild type HerA and HerA(D176E) could functionally complement *herA* deficiency *in vivo* under the assay conditions.

To gain insight into why only D176E could rescue *S. islandicus* cells with a chromosomal *herA* deletion, all six HerA mutant derivatives were expressed in and purified from *E. coli*. We showed that the wild type HerA was able to hydrolyze approximately 1.8 mole of ATP per mole of enzyme per min. The ATPase activity of HerA was enhanced in the presence of either ssDNA, 3′-overhang, or blunt-ended dsDNA (Figure 2C). In addition, the ATPase activity of HerA was not enhanced in the presence of NurA (Figure 2C). Except for D176E and E356D, the ATPase activity of all the remaining HerA mutants was very low or undetectable. D176E and E356D maintained about one-seventh and one-fifth the activity of the wild-type enzyme, respectively (Figure 2D). Notably, the ATPase-active mutant E356D could not rescue the chromosomal deficiency of HerA, which was further studied (see below). Taken together, these results indicate that ATPase activity is essential, but not sufficient on its own, for the *in vivo* function of HerA.

### Nuclease activity is essential for the *in vivo* function of NurA

**Figure 3:**
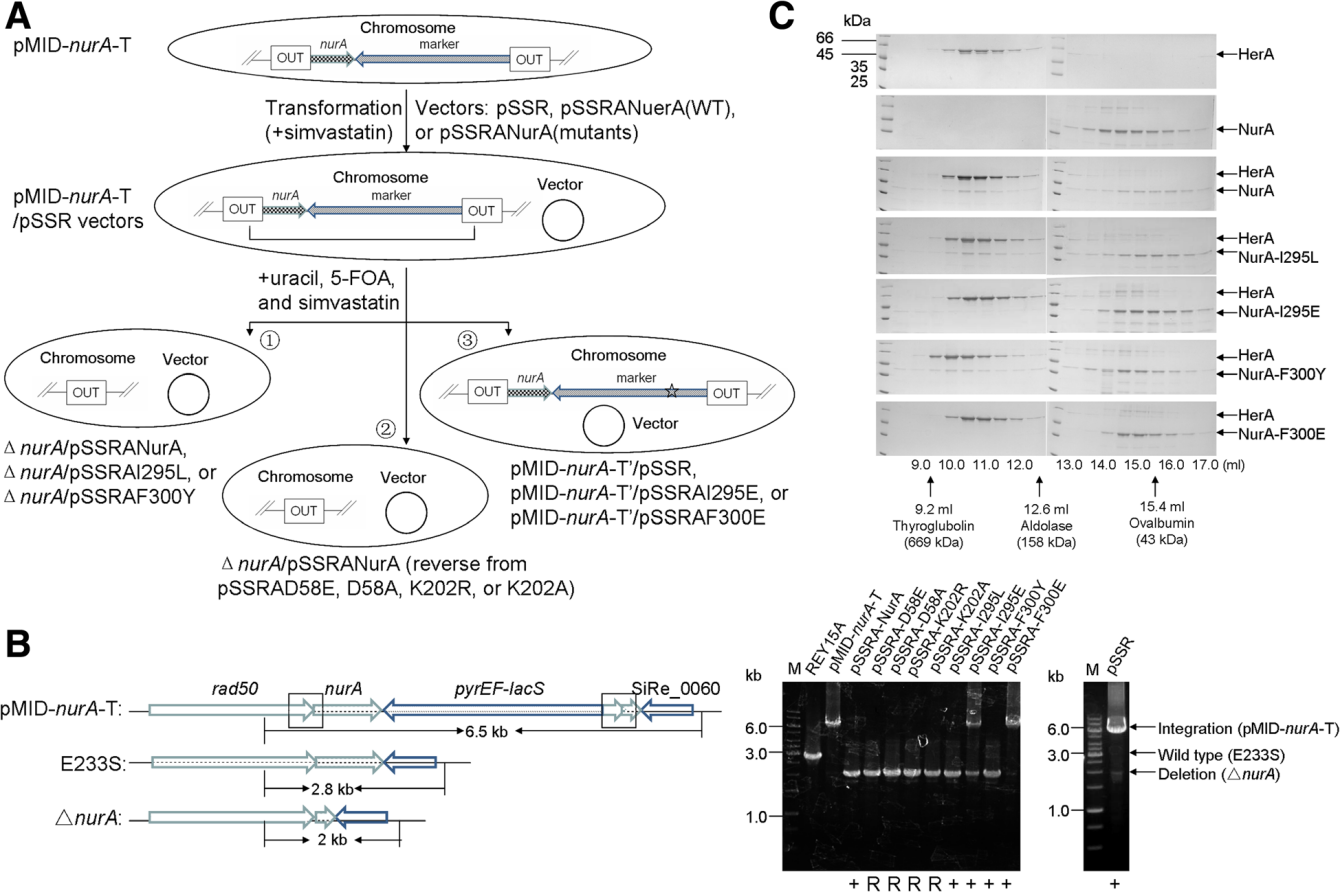
**The nuclease activity of NurA and its interaction with HerA are essential for cell viability. (A)** A schematic map depicting the procedures of the complementation analysis. pMID-*nurA*-T cells were transformed with pSSRA-NurA-C-His plasmids or empty vector. Three genotypes of the cells grown up after counter-selection were indicated as ➀, ➁, and ➂. The star indicates a putative spontaneous mutation in *pyrEF*. **(B)** Genotype analysis of the cells grown up by PCR using *nurA* flanking primers and sequencing of the *nurA* on the isolated plasmid. “+”, the designed plasmid maintained; “R”, plasmid maintained, but *nurA* gene had reverted to wild-type. **(C)** Gel filtration analysis of the interaction between wild type HerA and NurA mutants I295L, I295E, F300Y, and F300E by a Superdex™ 200 10/300 GL column. A total of 500 μg HerA, 500 μg NurA, and the mixture were heated incubated at 60°C for 20 min before gel filtration. The sample fractions were analyzed by SDS-PAGE. A small amount of NurA formed dimers in gels when NurA concentration was high. The elution peaks of the molecular markers are indicated by arrows at the bottom.

Previous works established that a few amino acid residues in NurA are critical for its enzyme activity. The corresponding residues are D58, E116, and D133 of *S. islandicus* NurA [26,44]. Another residue (K202 in *S. islandicus*) critical for the enzyme activity was revealed by crystallization analysis of *S. solfataricus* NurA. It is located near the active site of the NurA and substitution of the corresponding residue in *S. solfataricus* yielded an inactive enzyme, indicating that the lysine residue is possibly involved in the catalysis [26]. Since the NurA proteins from *S. solfataricus* and *S. islandicus* exhibit high identity (90%) in their amino acid sequences, the conserved residues should function in the same way. Therefore, we focused on two of the residues D58 and K202 and addressed the necessity of NurA nuclease activity using the same approach employed for HerA. The empty vector and pSSR carrying genes encoding the wild type NurA or its mutants D58E, D58A, K202R, and K202A were constructed and transformed into pMID-*nurA*-T cells. Surprisingly, all of the transformants grew under simvastatin selection, not only for the empty vector and pSSR carrying the wild type gene, but also for all the four mutants. After subsequent counter-selection by 5-FOA, colonies were selected from the plates and cultivated for further analyses. PCR analyses showed that the chromosomal *nurA* had been deleted in all strains supplemented with NurA (wild type or mutants), while that supplemented with the empty vector had not been deleted (Figure 3A and 3B).

Subsequently, *nurA* on the complementation plasmid was amplified and sequenced from each *nurA*-rescued strain. Intriguingly, all plasmids carried the wild-type *nurA* gene rather than any of the various mutant *nurA* genes cloned into the pSSR vector. This process was repeated three times, and the same results were obtained each time. Therefore, we conclude that the *nurA* mutants reverted to the wild-type during the experiment. Since reverse mutation occurs at a very low rate, we reasoned that the *nurA* mutant complementation plasmids could not rescue *nurA* deficiency in this archaeon. Furthermore, NurA and HerA work as a complex for dsDNA end processing in *S. solfataricus* and the NurA(K202A) mutant did not affect the interaction [26], suggesting that the inability to rescue *nurA* deficiency was not due to failure in the complex formation. Our results provide evidence supporting the hypothesis that nuclease activity is essential for the *in vivo* function of NurA. The reason why the plasmid carrying lethal NurA mutant existed in the cell but not for HerA mutants is not quite clear. We assume that the cells carrying plasmid with lethal NurA mutant have the possibility to assemble a functional NurA as a dimer, allowing the presence of the plasmid and the reversion to occur. While for the cells carrying plasmid with lethal HerA mutant, incorporation of a lethal deficient subunit into HerA hexamer could result in a non-functional HerA. As a result, the probability of assembling a functional HerA as a hexamer should be much lower than that for NurA as a dimer, and the cells would not likely survive.

### Interaction between NurA and HerA is essential to cell viability

A biochemical study has shown that in *S. solfataricus*, NurA interacts with HerA, forming a complex for dsDNA end processing [26]. Two NurA residues (I295 and F300), both found on a hydrophobic surface according to the protein’s crystal structure, have been implicated in the HerA-NurA interaction, as mutation at either residue to glutamic acid abolishes formation of the complex [26].

To address the importance of the HerA-NurA interaction *in vivo*, pSSR plasmids carrying four *S. islandicus* NurA mutants, I295L, I295E, F300Y, and F300E, were individually transformed into pMID-*nurA*-T cells to test for complementation. I295L and F300Y were able to achieve complementation (Figure 3B), whereas I295E and F300E failed to do so. Plasmid sequencing confirmed that each plasmid carried the original mutant *nurA* substitution. It is notable that the changes from isoleucine and phenylalanine to leucine and tyrosine (I295L and F300Y) do not affect the hydrophobic character at the interaction surface between NurA and HerA, whereas the I295E and F300E substitutions do. We speculated that NurA I295L and F300Y mutants should maintain the interaction, while I295E and F300E should not. To test this, the interaction between the HerA and NurA mutants I295L, I295E, F300Y, and F300E of *S. islandicus* were experimentally evaluated by gel filtration, confirming that I295L and F300Y maintained the interaction and complex formation while I295E and F300E did not (Figure 3C). It is interesting that the pSSR plasmid carrying NurA(I295E) or F300E was maintained in cells. We assume that I295E or F300E are not as toxic as the nuclease-dead NurA mutants (D58E, D58A, K202R, and K202A) since they were unable to interact with HerA and did not interfere with the DNA end resection process. The plasmid with either NurA mutant could exist in the cell and there is no selection for generation of the reversion genotype. Taken together, we speculate that the interaction between NurA and HerA is essential for cell viability.

### The HerA(D176E)-NurA complex, but not the HerA(E356D)-NurA complex, retains 5′-3′ DNA resection activity *in vitro*

**Figure 4:**
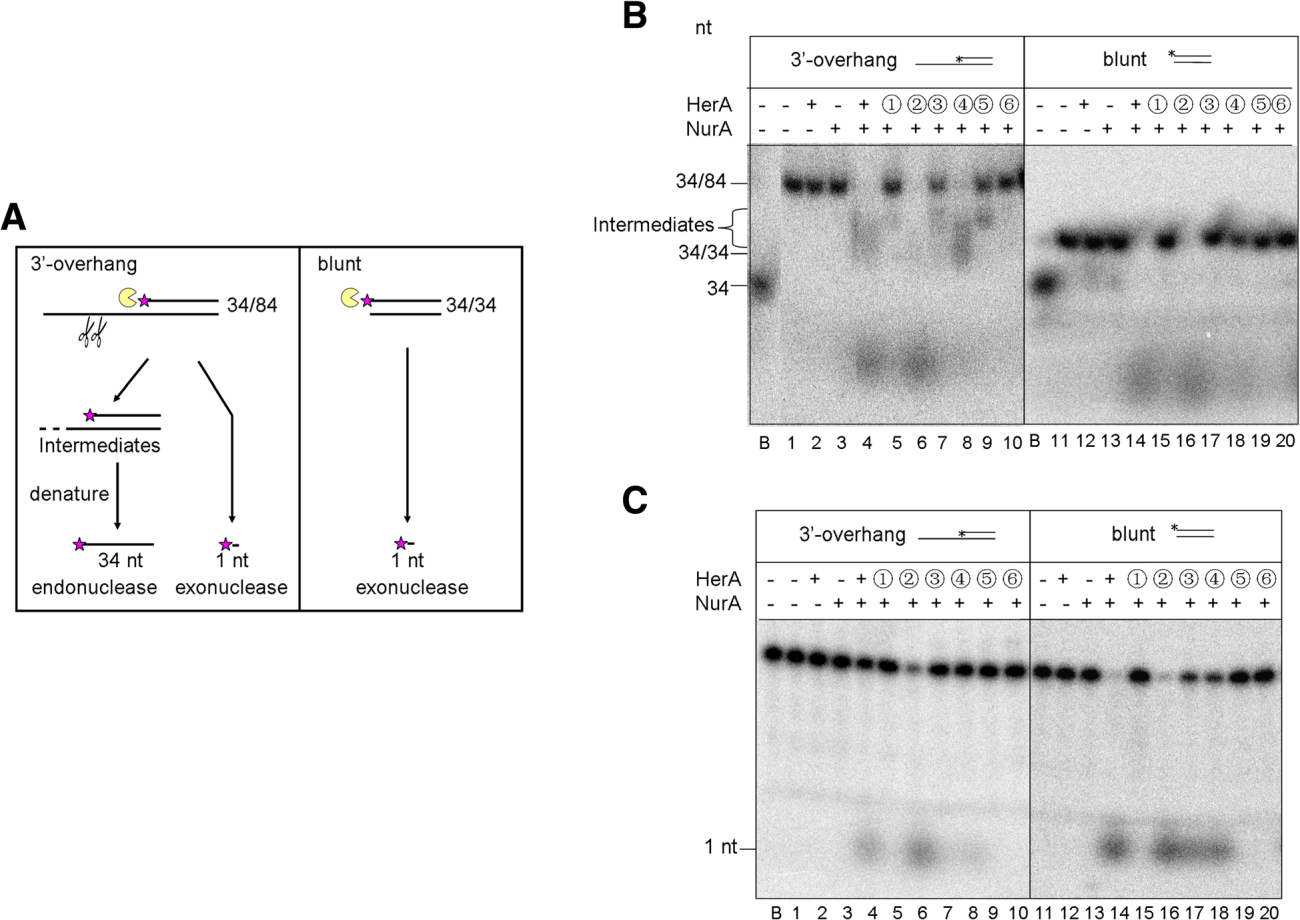
**HerA(D176E)-NurA maintains strong 5′-3′ exonuclease activity. (A)** A schematic diagram of the nuclease activity of the HerA-NurA complex using two different substrates. **(B)** Represent gel profiles showing the nuclease activity of HerA-NurA complexes. Wild-type or mutant HerA (27.8 nM hexamer) was mixed with NurA (83.4 nM dimer) for 30 min at 65°C. After the reactions, the products were analyzed on a 10% native polyacrylamide gel. **(C)** Same as in **(B)**, but the products were analyzed on a 15% denaturing polyacrylamide gel. “+”, wild-type HerA or NurA. ➀-➅,site-directed HerA mutants: K154R, D176E, D176N, E356D, E356Q, and R381K. **B**, boiled sample.

As described above, although HerA(E356D) showed a higher level of ATPase activity than D176E, only HerA(D176E) rescued *herA* deficiency. Here, we further characterized the two mutant proteins. The activities of HerA, NurA, and their complex were examined using a 3′-overhang or blunt-ended DNA as the substrate (Additional file 3: Table S3 and Additional file 5: Table S4). The substrates were labeled, as illustrated in Figure 4A and Additional file 5: Table S4, and products and substrates were analyzed by native as well as denaturing polyacrylamide gel electrophoresis. The native gels were used to investigate both endonuclease and exonuclease activities whereas denaturing gels were used to investigate exonuclease activity of the substrates.

**Figure 5:**
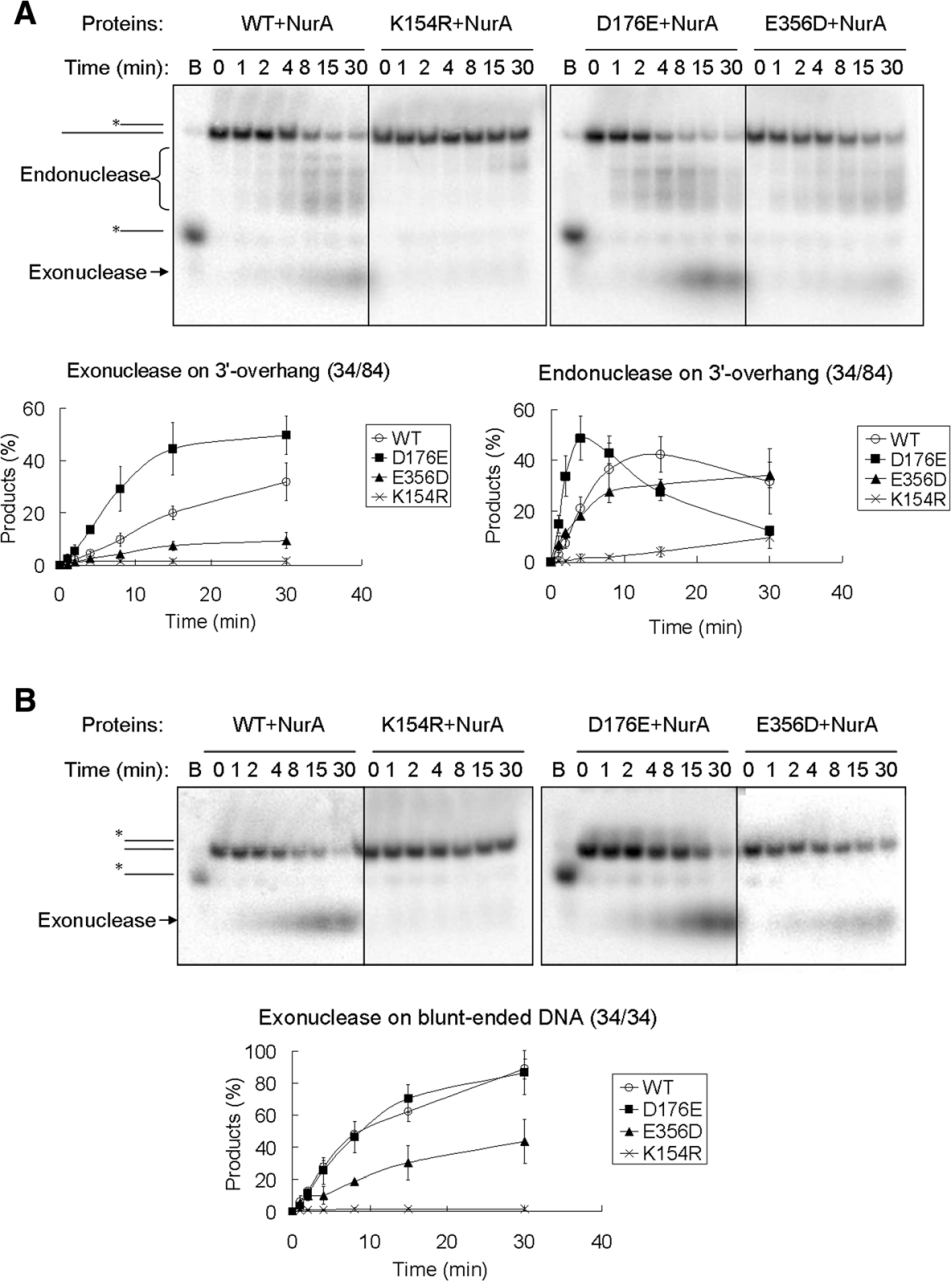
**The time courses of the exonuclease and endonuclease activities of HerA-NurA complexes on dsDNA.** The assay was performed in 20 μl reaction mixture as described in the Methods. The reactions were stopped at the indicating times by the addition of 5× loading buffer and putting the tubes on ice. The representative gels and the means ± standard deviations of three independent experiments are shown. **(A)** 3′-overhang. **(B)** blunt-ended DNA.

We first investigated the DNA degradation activity of the wild-type HerA and NurA. In the absence of ATP, the HerA-NurA complex failed to degrade any of the DNA substrates, indicating that the nuclease activity of NurA was dependent on the ATP hydrolysis by HerA (Additional file 7: Figure S1B). Furthermore, HerA also lost its helicase activity when it was alone or combined with nuclease-dead NurA protein (Additional file 7: Figure S1D). This indicates that the DNA degradation activity of HerA and NurA relies on both ATPase (and/or helicase) activity of HerA and the nuclease activity of NurA, in agreement with a previous report [26]. HerA-NurA complex degraded 3′-overhang and blunt-ended DNA in the 5′-3′ direction, although it also exhibited partial endonuclease activity on 3′-overhang DNA (Additional file 7: Figure S1B and S1C). Subsequently, since we were unable to detect the helicase activity of HerA in the absence of NurA, the DNA degradation activity of various HerA mutants complexed with wild-type NurA was analyzed (Figure 4). For blunt-ended substrates, the 5′-3′ exonuclease activity of D176E-NurA was similar to wild-type HerA-NurA (Figure 4B and 4C, lanes 14 and 16; Figure 5B). For 3′-overhang substrate, D176E-NurA exhibited higher exonuclease activity (Figure 4C, lanes 4 and 6; Figure 5A) and endonuclease activity (Figure 5A) than the wild-type HerA-NurA, while for both substrates, R381K-NurA were unable to degrade DNA. This was thought to be due to loss of ATPase activity (Figure 4B and 4C, lanes 10 and 20). K154R-NurA, D176N-NurA, and E356Q-NurA exhibited much lower activity than wild-type HerA-NurA or were completely inactive (Figure 4B and 4C, lanes 5, 7, 9, 15, 17, and 19; Figure 5). Importantly, we found E356D-NurA exhibited nuclease activities distinct from both wild-type HerA-NurA and HerA(D176E)-NurA. E356D-NurA maintained endonuclease activity on the 3′-overhang substrate but with less than half of the exonuclease activity of the wild type on any of the substrate (Figure 4B and 4C, lanes 8 and 18; Figure 5). Since we have shown that only HerA(D176E) rescued the deficiency of the chromosomal *herA* but HerA(E356D) did not, these results reveal, for the first time, that high 5′-3′ exonuclease activity of HerA-NurA is essential for cell viability.

### HerA interacts with NurA *in vivo*

**Figure 6:**
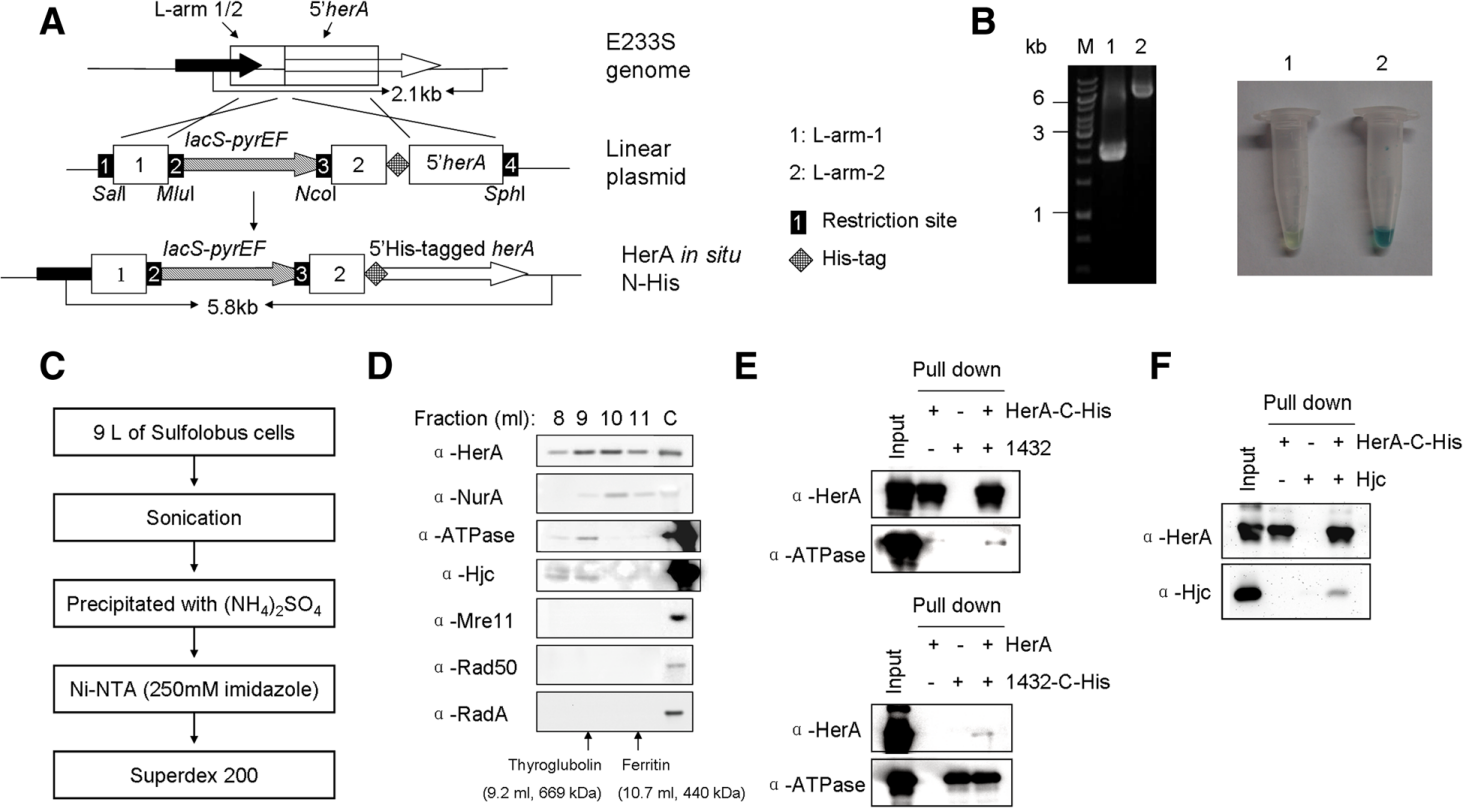
**Identification of co-purified proteins using a chromosomally-coded His-tagged HerA. (A)** Schematic diagram showing the construction of a strain that can encode a N-terminal His-tagged HerA by modification of the chromosomal *herA*. The MID strategy was used for the construction. **(B)** Confirmation of the constructed strain using PCR and X-gal staining. *herA* locus-specific flanking primers were used for PCR amplification. 1, the control strain E233S; 2, the constructed strain pMIDHis-*herA*-T. **(C)** Schematic map showing the purification of the proteins. **(D)** Western blot analyses of *in situ* N-His-tagged HerA and its co-purified proteins with specific antibodies. The fractions of 8, 9, 10, and 11 ml collected from the gel filtration were analyzed. C, purified protein. The standard molecular mass markers are indicated with arrows. **(E)** Pull-down assay confirming the interaction between HerA and the ATPase. A total of 50 μg HerA-C-His (or HerA) was mixed with 50 μg ATPase (or ATPase-C-His) at 70°C for 30 min in a buffer containing 25 mM Tris-HCl, pH 9.0, and 200 mM NaCl. The mixture was incubated with nickel-agarose beads. Unbound proteins were washed with the buffer above supplemented with 25 mM imidazole and target proteins were eluted by 250 mM imidazole. The fractions were detected by western blot with protein-specific antibodies. **(F)** Detection of the interaction between HerA and Hjc by pull-down assay. The procedure was as those used in **(E)**. A total of 42 μg HerA-C-His and 12 μg Hjc were used in the assay.

To further investigate the *in vivo* role of HerA, we conducted *in situ* poly-histidine (His)-tagged protein purification. This method facilitated the isolation of HerA-interacting proteins in *S. islandicus* cells under physiological growth conditions. Using a recombination scheme similar to marker insertion and target gene deletion (MID), the chromosomal version of *herA* was replaced with an N-His-tagged version of *herA* (Figure 6A). The resulting strain was confirmed by PCR using *herA* locus-specific flanking primers and X-gal staining (Figure 6B). His-tagged HerA and associated proteins were co-purified from cell extract of a 9-L culture by ammonium sulfate precipitation, nickel-nitrilotriacetic acid (Ni-NTA) affinity purification, and gel filtration (Figure 6C). Purified proteins were analyzed by SDS-PAGE and Western blotting. Western blot analysis using protein-specific antibodies revealed that NurA eluted in same fractions with HerA (Figure 6D). The fractions containing HerA were pooled, concentrated, and analyzed further by liquid chromatography-mass spectrometry. Strikingly, we identified two other proteins co-purified with HerA, Hjc (Holliday junction cleavage, SiRe_1431) and a PIN-domain ATPase (SiRe_1432). Western blot analysis showed that HerA, Hjc, and the PIN-domain ATPase were present in earlier fractions of the co-purified sample than that of HerA and NurA (Figure 6D). However, the HRR proteins, Mre11, Rad50, and RadA, were not found in either MS (data not shown) or Western blot analyses (Figure 6D). The interaction between HerA and the PIN-domain ATPase or Hjc may be weak in the absence of other factors as shown in an *in vitro* pull-down assay (Figure 6E and 6F). The results support that HerA forms a complex with NurA *in vivo* and suggest that HerA has additional partners in the cell.

## Discussion

### Essentiality of the *mre11* operon and the 5′-3′ exonuclease activity of the HerA-NurA complex

Previous works suggested that each gene in the *mre11* operon could be essential in *S. islandicus* and *T. kodakaraensis.* However, the gene essentiality in *T. kodakaraensis*, an archaeon belonging to euryarchaeota, one of the two main branches of archaea, was deduced from the inability of isolating mutants of respective genes whereas only an overview has been presented for the genetic study for the HRR genes in *S. islandicus*, an archaeon belonging to crenarchaeota, another main branch of archaea [29,32,33]. Here, we have evaluated the gene essentiality of the four genes in *S. islandicus* individually, using a mutant propagation assay previously developed in our laboratory [38]. We found that mutant cells generated from the pMID transformants (pMID-T) lost their propagation capability, strongly demonstrating that each of the genes in this operon is essential for cell viability of *S. islandicus*. Our previous studies have revealed that a RecQ-like DNA helicase Hjm and RadA, the archaeal homolog of Rad51, are essential in this archaeon [32,33]. These results indicate that all these HRR genes are essential for the viability of *S. islandicus*. Strikingly, hyperthermophilic archaea are the only known organisms among microorganisms, in which the *mre11*, *rad50*, and *radA* are absolutely required for cell growth.

We attempted to quantify the levels of HerA and NurA in the cell by quantitative Western blot analysis and found that the amounts were about 956 ± 30 and 848 ± 40 molecules per cell for HerA and NurA, respectively (Additional file 8: Figure S2). These are much higher than that for RecBCD, a DNA end resection complex in *E. coli*, which is only about 10 molecules per cell [45]. This may indicate that a large number of DSBs exist in *Sulfolobus* which need to be repaired constantly and efficiently. In response to the essential requirement of DSB processing, HerA and NurA may be constitutively expressed in high levels in the cells.

The essentiality of the activities of the HRR proteins in this organism was further characterized using a genetic complementation assay recently developed in our laboratory [34]. We have found that the ATPase activity of HerA and the efficient 5′-3′ degradation activity by the HerA and NurA complex is essential for cell viability in *S. islandicus* and this should reflect the *in vivo* activity of the enzyme complex. We have observed that HerA(D176E) have high exonuclease activity and could maintain the essential function of HerA *in vivo*. Another mutant (E356D) of HerA and NurA wild-type protein complex maintain the ssDNA endonuclease activity of the wild type enzyme complex, but with much reduced exonuclease activity. We have shown that HerA(E356D) did not complement the essential function of the protein complex and therefore the endonuclease activity apparently does not reflect the *in vivo* essentiality of the helicase/nuclease complex; however, the exonuclease to generate ssDNA does. The ssDNA generated during DSB end resection is a critical intermediate for strand invasion in eukaryote. It could also be utilized in a synthesis-dependent strand-annealing pathway (SDSA) [46]. Therefore, although the ssDNA is mostly likely employed in HRR in archaea, the possibility that it is a substrate for other processes such as a putative SDSA can not be ruled out.

### Roles of the conserved residues of HerA

We revealed that out of the six HerA mutants, only HerA(D176E) could maintain the essential function of HerA *in vivo*. Both HerA(K154R) and HerA(R381K) lost the ATPase activity of HerA, confirming the importance of the Walker A and arginine finger motif in ATP hydrolysis for DNA helicase [47-49]. It was recently shown that E356 of the Walker B motif in *S. solfataricus* HerA forms a salt bridge with trans-acting R381. This interaction is thought to be necessary for both stable interface formation and water activation prior to catalysis [43]. The change of glutamate acid (E) to glutamine (Q) may impair the formation of the salt bridge leading to loss of the ATPase activity. Furthermore, HerA(E356D)-NurA maintained the same level of the endonuclease activity as the wild type, but had much lower 5′-3′ exonuclease activity on dsDNA. E365 may be also involved in fine coupling of ATP hydrolysis and DNA unwinding. Slight change of the residue from glutamic acid to aspartic acid could lead to coupling impairment resulting in the reduction of the 5′-3′ resection of HerA(E356D)-NurA. In agreement with this, *S. solfataricus* HerA structure showed that ATP binding, hydrolysis, and release led to the local changes in the positioning of R381, which resulted in DNA-binding loop movement and substrate translocation [43]. The E356D mutant may maintain the salt bridge with R381 for ATP binding and hydrolysis, but affect the changes of R381 positioning, which would impair its DNA unwinding activity. Finally, we showed that HerA(D176N) exhibited as low ATPase activity as that of E356Q. The conserved residue D176 in *S. solfataricus* HerA locates close to the ATP-binding site. Our results suggest that mutation to either glutamic acid or asparagine affects the ATP activity, and is in good agreement with the structural prediction of HerA.

### The end resection activity requires both HerA and NurA

It is surprising that we could not detect the helicase activity of HerA or the nuclease activity of NurA, even though various reaction conditions were applied in our assay. The DNA end resection activity on dsDNA was only observed in the presence of both HerA and NurA (Additional file 7: Figure S1B and S1D). The result was consistent with that in *S. solfataricus* and *P. furiosus* [23,26]. However, the helicase activity of HerA and the nuclease activity of NurA could be detected using the HerA and NurA proteins from *S. tokodaii* and *S. acidocaldarius* [18,19,22,44]. Due to the closer phylogenetic relationship of *S. islandicus* to *S. solfataricus* than that to either *S. tokodaii* or *S. acidocaldarius*, it may be reasonable that the properties of *S. islandicus* HerA and NurA proteins resemble to their counterparts in *S. solfataricus*. It may indicate that the mode of end resection by HerA and NurA in *S. islandicus* and *S. solfataricus* is different from that of *S. tokodaii* and *S. acidocaldarius*. Further study on how HerA interacts with NurA for dsDNA end resection and how the length of the resected ssDNA is controlled will reveal the detailed mechanisms of DNA end processing in archaea.

## Conclusion

Our results revealed that the high 5′-3′ DNA end resection activity of HerA-NurA complex is essential for cell viability. This may indicate that a large number of DSBs occurs in *Sulfolobus* cells under high temperature conditions need to be efficiently resected. Two other HerA-interacting proteins, an ATPase and Hjc, were identified, suggesting that HerA have other roles in addition of that in DNA end resection. These results will help better understand HRR in thermophilic archaea.

## Additional files

Additional file 1: Table S1. Strains used in this study. Additional file 2: Table S2. Plasmids used in this study. Additional file 3: Table S3. Oligonucleotides used in this study. Additional file 4:
**Supplementary methods.**
Additional file 5: Table S4. Substrates used in this study. Additional file 6: Table S5. Peptides for making protein-specific antibodies used in this study. Additional file 7: Figure S1. Both HerA and NurA are required for DNA degradation. (A) A schematic illustrating nuclease activity analysis. (B) Analysis of DNA degradation of 3′-overhangs and blunt-ends in the presence and absence of ATP by a HerA-NurA mixture. HerA (27.8 nM hexamer) was mixed with NurA (83.4 nM dimer) and incubated at 65°C for 30 min. Samples were analyzed on a 10% native polyacrylamide gel. B, boiled samples. (C) Same as in (B), but the samples were analyzed on a 15% denaturing polyacrylamide, and only samples with ATP were analyzed. M, size marker. (D) A HerA-NurA complex containing a nuclease inactive form of NurA (D58A) loses DNA degradation activity. B, boiled samples. The concentrations of HerA and NurA(D58A) used for 3′-overhang substrates were 0.0625, 0.125, 0.25, 0.5, 1, and 2 μM. The concentrations of HerA and NurA(D58A) used for blunt-ended DNA substrates were 0.125, 0.25, 0.5, and 1 μM. Additional file 8: Figure S2. Determination of HerA and NurA amounts in *S. islandicus* cells. Total proteins of wild type cells were separated by SDS-PAGE for quantitative Western blot. Purified HerA (19 ng) and NurA (45 ng) proteins from *E. coli* were loaded as standards for quantification.

## Abbreviations

DSBs: Double-strand breaks
HRR: Homologous recombination repair
NHEJ: Non-homologous end joining
SDSA: Synthesis-dependent strand-annealing pathway
5-FOA: 5-fluorotic acid
MID: Marker insertion and target gene deletion
