# Supplementary material for: Efficient 5′-3′ DNA end resection by HerA and NurA is essential for cell viability in the crenarchaeon Sulfolobus islandicus
Source: BMC Mol Biol. 2015 Feb 14;16:2. doi: 10.1186/s12867-015-0030-z (PMC4351679; doi:10.1186/s12867-015-0030-z)
Supplement: Additional file 1: Table S1. — Strains used in this study. [file 12867_2015_30_MOESM1_ESM.doc]

**Additional file 1: Table S1. Strains used in this study**

| **Strains** | **Genotype** | **Source or reference** |
| --- | --- | --- |
| *S. islandicus* REY15A | Wild type | Contursi, *et al*., 2006. |
| *S. islandicus* REY15A (E233S) | Δ*pyrEF*Δ*lacS* | Deng, *et al*., 2009. |
| pMID-*herA*-T, pMID-*mre11*-T, pMID-*rad50*-T, pMID-*nurA*-T, pMID-*orc1-3*-T | E233S integrated transformant generated by double-crossover with pMID-*herA*, pMID-*mre11*, pMID*-rad50,* pMID-*nurA*, or pMID-*orc1-3*, respectively | This work and Zheng, *et al*., 2012. |
| Δ*herA*/pSSRA-HerA-C-His | Δ*herA* with *herA* (C-His) on the complementing vector pSSR harboring *araS* promoter | This work |
| pMID-*herA*-T/pSSRA-HerAD176E-C-His | pMID-*herA*-T with *herA*D176E on the complementing vector pSSR harboring *araS* promoter | This work |
| Δ*herA* /pSSRA-HerAD176E-C-His | Δ*herA* with *herA*D176E on the complementing vector pSSR harboring *araS* promoter | This work |
| Δ*nurA*/pSSRA-NurA-C-His,  Δ*nurA*/pSSRA-NurAI295L-C-His,  Δ*nurA*/pSSRA-NurAF300Y-C-His | Δ*nurA* with *nurA,* *nurA*I295L, or *nurA*F300Y on the complementing vector pSSR harboring *araS* promoter | This work |
| pMID-*nurA*-T/pSSRA-NurAI295L-C-His, pMID-*nurA*-T/pSSRA-NurAI295E-C-His, pMID-*nurA*-T/pSSRA-NurAF300Y-C-His, pMID-*nurA*-T/pSSRA-NurAF300E-C-His | pMID-*nurA*-T with *nurA*I295L, I295E, F300Y, or F300E on the complementing vector pSSR harboring *araS* promoter | This work |
| pMIDHis-*herA*-T | E233S integrated transformant generated by double-crossover with pMIDHis-*herA*, | This work |
