# Supplementary material for: Efficient 5′-3′ DNA end resection by HerA and NurA is essential for cell viability in the crenarchaeon Sulfolobus islandicus
Source: BMC Mol Biol. 2015 Feb 14;16:2. doi: 10.1186/s12867-015-0030-z (PMC4351679; doi:10.1186/s12867-015-0030-z)
Supplement: Additional file 2: Table S2. — Plasmids used in this study. [file 12867_2015_30_MOESM2_ESM.doc]

**Additional file 2: Table S2. Plasmids used in this study**

| **Plasmids** | **Features** | **Source or reference** |
| --- | --- | --- |
| pMID-*herA* | pUC19 carrying L-arm, R-arm, and G-arm of *herA* gene of *S. islandicus* and the marker cassette (*pyrEF+lacS*) | Zheng, *et al*., 2012. |
| pMID-*mre11* | pUC19 carrying L-arm, R-arm and G-arm of *mre11* gene plus the marker cassette | This work |
| pMID-*rad50* | pUC19 carrying L-arm, R-arm and G-arm of *rad50* gene plus the marker cassette | This work |
| pMID-*nurA* | pUC19 carrying L-arm, R-arm and G-arm of *nurA* gene plus the marker cassette | This work |
| pSeSD | *E. coli/Sulfolobus* shuttle vector harboring the marker *pyrEF* | Peng, *et al*., 2012. |
| pSeSDA-N-His-HerA | pSeSD carrying *herA* genes with *araS* promoter coding for N-His-HerA | This work |
| pSSR | *E. coli/Sulfolobus* shuttle vector harboring the marker *sim^R^* | Zheng, *et al*., 2012. |
| pSSRA-HerA-C-His | pSSR carrying *herA* genes with *araS* promoter coding for HerA-C-His | This work |
| pSSRA-HerAK154R-C-His,  pSSRA-HerAD176E-C-His,  pSSRA-HerAD176N-C-His,  pSSRA-HerAE356D-C-His,  pSSRA-HerAE356Q-C-His,  pSSRA-HerAR381K-C-His | pSSR carrying *herA*K154R, D176E, D176N, E356D, E356Q, or R381K gene with *araS* promoter | This work |
| pSSRA-NurA-C-His, | pSSR carrying *nurA* gene with *araS* promoter | This work |
| pSSRA-NurAD58A-C-His,  pSSRA-NurAD58E-C-His,  pSSRA-NurAK202A-C-His,  pSSRA-NurAK202R-C-His,  pSSRA-NurAI295E-C-His,  pSSRA-NurAI295L-C-His,  pSSRA-NurAF300E-C-His,  pSSRA-NurAF300Y-C-His | pSSR carrying *nurA*D58A, D58E, K202A, K202R, I295E, I295L, F300E, or F300Y with *araS* promoter | This work |
| pET29a-HerA-C-His | pET29a carrying *herA* | This work |
| pET29a-HerAK154R-C-His,  pET29a-HerAD176E-C-His,  pET29a-HerAD176N-C-His,  pET29a-HerAE356D-C-His,  pET29a-HerAE356Q-C-His,  pET29a-HerAR381K-C-His | pET29a carrying *herA*K154R, D176E, D176N, E356D, E356Q, or R381K | This work |
| pET29a-NurA-C-His | pET29a carrying *nurA* | This work |
| pET29a-NurAD58A-C-His, pET29a-NurAI295L-C-His, pET29a-NurAI295E-C-His, pET29a-NurAF300Y-C-His, pET29a-NurAF300E-C-His | pET29a carrying *nurA*D58A, I295E, I295L, F300E, or F300Y | This work |
| pMIDHis-*herA* | pUC19 carrying L-arm-1, L-arm-2 and G-arm of 5’ herA gene plus the marker cassette | This work |
