# Supplementary material for: Efficient 5′-3′ DNA end resection by HerA and NurA is essential for cell viability in the crenarchaeon Sulfolobus islandicus
Source: BMC Mol Biol. 2015 Feb 14;16:2. doi: 10.1186/s12867-015-0030-z (PMC4351679; doi:10.1186/s12867-015-0030-z)
Supplement: Additional file 3: Table S3. — Oligonucleotides used in this study. [file 12867_2015_30_MOESM3_ESM.doc]

**Additional file 3: Table S3. Oligonucleotides used in this study**

| **Primer** | **Sequence ^a^ (5’-3’)** |
| --- | --- |
| mre11-IN-F-XhoI | TAGT**CTCGAG**TCAATGGATGCAGTAGTCTCA |
| mre11-IN-R-MluI | GACT**ACGCGT**TACCATCATCAATCACCAATT |
| mre11-OUT-F-MluI | GATA**ACGCGT**GAGATATTAAAAAGACTAACAGGTG |
| mre11-OUT-R-SalI | GTCACG**GTCGAC**TCCCCATAATGTCTTGTAAAT |
| mre11-Tg-F-PstI | TGAAATA**CTGCAG**ATGGTACAAATTCTACATATTTCTG |
| mre11-Tg-R-SphI | TCAG**GCATGC**AAAGCATAATACCCAAAACCT |
| rad50-IN-F-XhoI | ATCT**CTCGAG**AGGTTTTGGGTATTATGCTTT |
| rad50-IN-R-MluI | GATT**ACGCGT**TCTCATAAGTTAACACCTGTTAGTC |
| rad50-OUT-F-MluI | GAGT**ACGCGT**AGTTTTGTGAGGGAGGAGACTT |
| rad50-OUT-R-SalI | GTATG**GTCGAC**ATTCTTAACTAGCTTTAGCTCTTC |
| rad50-Tg-F-PstI | TCAATA**CTGCAG**ATGAGAATTGACAAAATTACTTT |
| rad50-Tg-R-SphI | ATGC**GCATGC**TTTATCTCATCCTCCAGTTTT |
| nurA-IN-F-MluI | GACT**ACGCGT**TATTAATCGTCATCATTCGTCG |
| nurA-IN-R-XhoI | TACA**CTCGAG**GGGAGGCTTTCTTATGGATT |
| nurA-OUT-F-SalI | CTAAC**GTCGAC**ACACTAATAACGGCGATACTT |
| nurA-OUT-R-MluI | GATC**ACGCGT**TTTACTGAAAGAACCACCATC |
| nurA-Tg-F-SphI | TGAC**GCATGC**AAAAGATGAAAGGAAAATGAGG |
| nurA-Tg-R-PstI | TCAAGT**CTGCAG**TTAATAAAACTGGCTGGGC |
| herA-F-NdeI | CGCCG**CATATG**ATAATTGGTTATGTAATTGGTC |
| herA-R-SalI | CTA**GTCGAC**ATCACCAATTTCCGTTCCAAAG |
| herA-R-XhoI | CTAT**CTCGAG**TTAATGGTGGTGATGATGATG |
| herA-F-ClaI | CGGC**ATCGAT**ATAATTGGTTATGTAATTGGTCAAG |
| herA-NoHis-R-SalI | CGTC**GTCGAC**TCAATCACCAATTTCCGTTC |
| HerAK154R-R | GCTACTGTATTTGACCTCCCAGAACCA |
| HerAK154R-F | TGGTTCTGGGAGGTCAAATACAGTAGC |
| HerAD176E-R | GTACTCTCCATGATACTCAAAGATAAGAACAG |
| HerAD176E-F | CTGTTCTTATCTTTGAGTATCATGGAGAGTAC |
| HerAD176N-R | GTACTCTCCATGATAATTAAAGATAAGAACAG |
| HerAD176N-F | CTGTTCTTATCTTTAATTATCATGGAGAGTAC |
| HerAE356D-R | GAAAACGTGAGCATCTTCTATTACGGC |
| HerAE356D-F | GCCGTAATAGAAGATGCTCACGTTTTC |
| HerAE356Q-R | GAAAACGTGAGCTTGTTCTATTACGGC |
| HerAE356Q-F | GCCGTAATAGAACAAGCTCACGTTTTC |
| HerAR381K-R | CAACTCCGAATTTTTTGCCCTCTCTTG |
| HerAR381K-F | CAAGAGAGGGCAAAAAATTCGGAGTTG |
| nurA-F-NdeI | GCCTCGCC**CATATG**ATAAGAAAAATATATGAT |
| nurA-R-NotI | TAATGATA**GCGGCCGC**ATAAAACTGGCT |
| NurAD58A-R | CTGAAAGAACCACCAGCAATAGCTACAAATT |
| NurAD58A-F | AATTTGTAGCTATTGCTGGTGGTTCTTTCAG |
| NurAD58E-R | CTGAAAGAACCACCTTCAATAGCTACAAATT |
| NurAD58E-F | AATTTGTAGCTATTGAAGGTGGTTCTTTCAG |
| NurAK202A-R | CCCCTACTGGTTGCAGATATCCATAAAAC |
| NurAK202A-F | GTTTTATGGATATCTGCAACCAGTAGGGG |
| NurAK202R-R | CCCCTACTGGTTCTAGATATCCATAAAAC |
| NurAK202R-F | GTTTTATGGATATCTAGAACCAGTAGGGG |
| NurAI295E-R | TGGATATCCCTTTTCGCTTACTCCACTC |
| NurAI295E-F | GAGTGGAGTAAGCGAAAAGGGATATCCA |
| NurAI295L-R | TGGATATCCCTTTAAGCTTACTCCACTC |
| NurAI295L-F | GAGTGGAGTAAGCTTAAAGGGATATCCA |
| NurAF300E-R | CTTTTAAGAGTGGTTCTGGATATCCCTTAATG |
| NurAF300E-F | CATTAAGGGATATCCAGAACCACTCTTAAAAG |
| NurAF300Y-R | CTTTTAAGAGTGGATATGGATATCCCTTAATG |
| NurAF300Y-F | CATTAAGGGATATCCATATCCACTCTTAAAAG |
| HerA For | CTTTTCCACCATGTCCTACATAA |
| HerA Rev | CACTACGTGCAACTGGTATTTG |
| NurA For | AACGAGTTTAGATGTCCATAATG |
| NurA Rev | CCCTATCTCTAAACTTCAAAACC |
| 5’HerA L-arm-1-SalI F | GCAG**GTCGAC**GGTTGCAATGACAGCAAAAC |
| 5’HerA L-arm-1-MluI R | CGCG**ACGCGT**GTATAGATAAAATACTATTGGCTTC |
| 5’HerA L-arm-2-NcoI F | CATG**CCATGG**GGTTGCAATGACAGCAAAAC |
| 5’HerA L-arm-2-6His R | GATGGTGGTGATGCATGTATAGATAAAATACTATTGGC |
| 5’-6His-HerA-G-arm F | GTATTTTATCTATACATGCATCACCACCATCATCAC |
| 5’HerA-G-arm-SphI R | CAGC**GCATGC**CCACCAAGTTCAGAAATTCT |
| Strand B (84-mer) | TCCTCTAGAGTCGACCTGCAGGCATGCAAGCTTGGCACTGGCCGTCCTTTTACAACGTCGTGACTGGGAAAACCCTGGCGTTAC |
| Strand E (34-mer) | CAAGCTTGCATGCCTGCAGGTCGACTCTAGAGGA |
| Strand F (34-mer) | TCCTCTAGAGTCGACCTGCAGGCATGCAAGCTTG |

^a^ Restriction sites are indicated in boldface and mutated codons for construction of site-directed mutants are underlined.
