# Supplementary material for: Efficient 5′-3′ DNA end resection by HerA and NurA is essential for cell viability in the crenarchaeon Sulfolobus islandicus
Source: BMC Mol Biol. 2015 Feb 14;16:2. doi: 10.1186/s12867-015-0030-z (PMC4351679; doi:10.1186/s12867-015-0030-z)
Supplement: Additional file 4: — Supplementary methods. [file 12867_2015_30_MOESM4_ESM.doc]

**Additional file 4: Supplementary Methods**

**Construction of plasmids for genetic complementation**

To construct HerA (C-termianl His-tagged) genetic complementation plasmids, *herA* was amplified by PCR using primers herA-F-*Nde*I and herA-R-*Sal*I. The PCR product was digested with the restriction enzymes and ligated into the *Nde*I and *Sal*I sites of the vector pSSR carrying a simvastatin-resistant marker *hmg*, yielding pSSRA-HerA-C-His.

For construction of NurA genetic complementation plasmid, the *nurA* gene was amplified using the genomic DNA and cloned into the *Nde*I and *Not*I sites of pSSR, generating plasmid pSSRA-NurA-C-His. The *herA* and *nurA* site-directed mutant fragments were obtained by splice overlap extension (SOE) PCR using their corresponding primers listed in Table S3 and cloned into pSSR as for the wild type *herA* and *nurA*, respectively.

**Construction of plasmids for protein expression in *E. coli***

The gene *herA* and those for its site-directed mutants were amplified by PCR using primers herA-F-*Nde*I and herA-R-*Xho*I and the corresponding pSSR plasmids carrying *herA* or its mutant genes and cloned into the *Nde*I and *Xho*I sites of the pET29a vector (EMD Millipore, Billerica, MA, USA). The fragments containing the *nurA* and its site-directed mutants were amplified using the pSSR plasmids and cloned into the *Nde*I and *Not*I sites of pET29a, generating pET29a-NurA-C-His that allows the expression of target proteins with C-terminal His-tag.

**Protein purification**

The pET29a plasmids carrying *herA*, *nurA*, or their mutant genes were transformed into *E. coli* BL21 (DE3)-CodonPlus-RIL for expression. IPTG (Merck, Darmstadt, Germany) was added for induction of proteins when OD_600_ of cells reached 0.2–0.6. After incubation at 37^o^C for further 4 hrs, cells were collected, resuspended in buffer A (50 mM Tris-HCl, pH 8.0, and 100 mM NaCl) and disrupted by sonication. Cell extract was heat-treated at 70^o^C for 30 min. Insoluble material was removed by centrifugation at 11,000 rpm for 15 min. For HerA, the supernatant was filtered with a 0.2-μm Millex-GP filter unit (Millipore, Billerica, MA, USA) and loaded onto a Ni-NTA column that had been pre-equilibrated with buffer A. The column was washed with six volumes of wash buffer (50 mM Tris-HCl, pH 8.0, 100 mM NaCl, and 30–40 mM imidazol). The target proteins were eluted with elute buffer (50 mM Tris-HCl, pH 8.0, 100 mM NaCl, and 250–300 mM imidazol). The eluted proteins were diluted in buffer A and concentrated before purification with a HiTrap Q FF column (GE Healthcare, Buckinghamshire, UK) that had been pre-equilibrated with buffer A. The proteins were eluted with a linear gradient of 0 to 1 M NaCl. The fractions containing target proteins (in 350–500 mM NaCl) were pooled, concentrated, and dialyzed against buffer A. The proteins were subsequently purified by gel filtration using a Superdex^TM^ 200 10/300 column (GE Health, UK) in buffer A. The purification of NurA is the same as that of HerA except that it was purified by a HiTrap^TM^ Heparin HP column (GE Health, UK) instead of a HiTrap Q FF column after Ni-NTA purification.
