# Supplementary material for: Efficient 5′-3′ DNA end resection by HerA and NurA is essential for cell viability in the crenarchaeon Sulfolobus islandicus
Source: BMC Mol Biol. 2015 Feb 14;16:2. doi: 10.1186/s12867-015-0030-z (PMC4351679; doi:10.1186/s12867-015-0030-z)
Supplement: Additional file 5: Table S4. — Substrates used in this study. [file 12867_2015_30_MOESM5_ESM.doc]

**Additional file 5: Table S4. Substrates used in this study**

| **Substrates** | **DNA structures ^a^** | **Oligonucleotides ^b^** |
| --- | --- | --- |
| 3’-overhang | 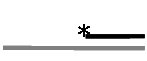 | E^﹡^+B |
| Blunt-ended DNA | 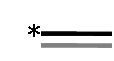 | E^﹡^+F |

^a^ Oligonucleotides labeled with ^32^P at 5’ end are indicated with an asterisk. Black lines indicate labeled strands and grey lines indicate unlabeled strands.

^b^ Oligonucleotide sequences are given in Table S3.
