# Supplementary material for: Efficient 5′-3′ DNA end resection by HerA and NurA is essential for cell viability in the crenarchaeon Sulfolobus islandicus
Source: BMC Mol Biol. 2015 Feb 14;16:2. doi: 10.1186/s12867-015-0030-z (PMC4351679; doi:10.1186/s12867-015-0030-z)
Supplement: Additional file 6: Table S5. — Peptides for making protein-specific antibodies used in this study. [file 12867_2015_30_MOESM6_ESM.doc]

**Additional file 6: Table S4. Peptides for making protein-specific antibodies used in this study**

| Antibody | Peptide sequence |
| --- | --- |
| α-HerA | SDNLSEDLAEQLSSC |
| α-Mre11 | CSIPGDHDTPKRKGY |
| α-Rad50 | CVEKRGNKSFVREET |
| α-NurA | TSRGRDLFGTDYPDC |
| α-RadA | CVTSHFRAEYPGREN |
| α-ATPase | DSKKGEANELSREYC |
| α-Hjc | NAKKPKGSAVERNIC |
