# Supplementary figures and images for: Efficient 5′-3′ DNA end resection by HerA and NurA is essential for cell viability in the crenarchaeon Sulfolobus islandicus
Source: BMC Mol Biol. 2015 Feb 14;16:2. doi: 10.1186/s12867-015-0030-z (PMC4351679; doi:10.1186/s12867-015-0030-z)

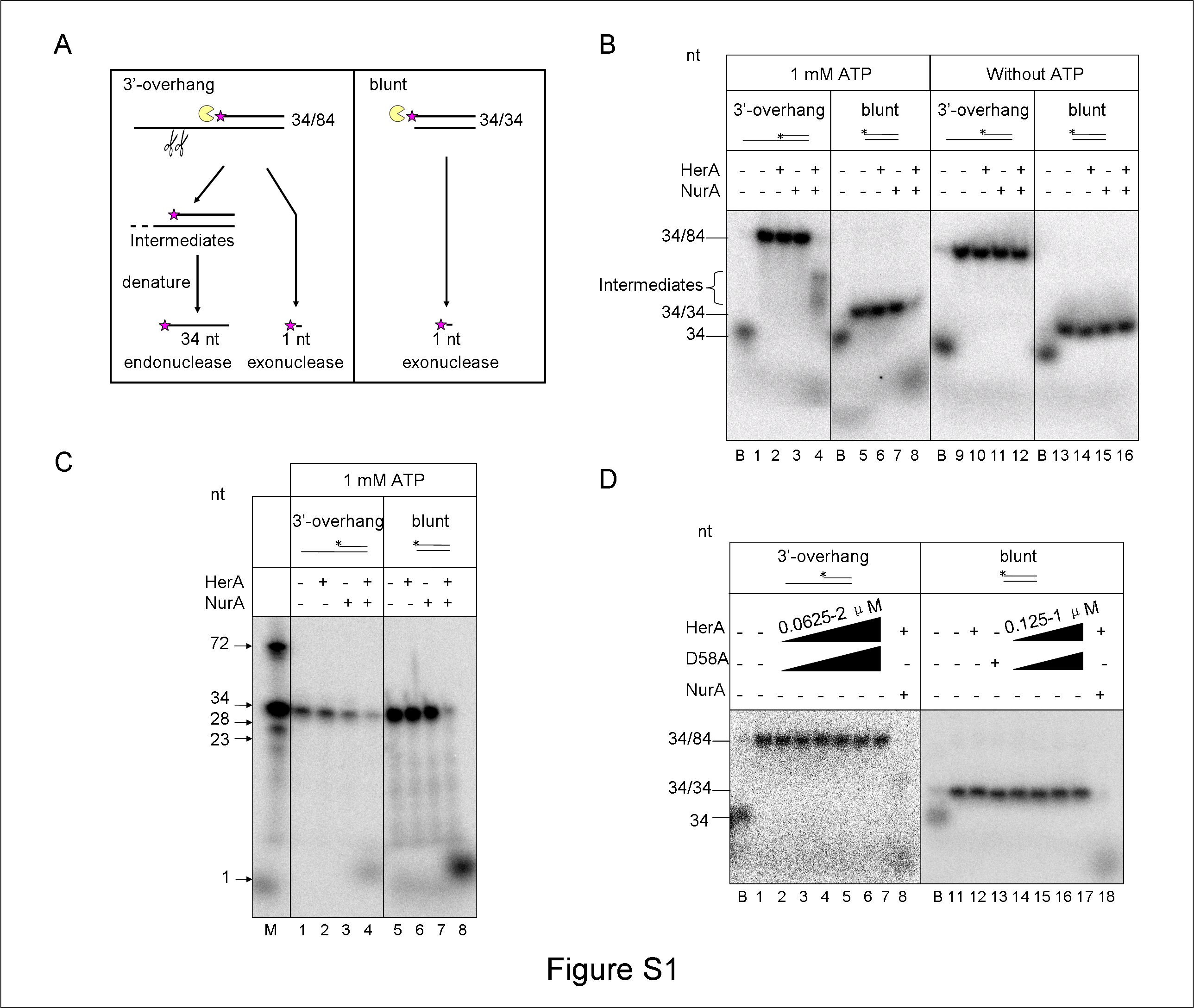

Supplement: Additional file 7: Figure S1. — Both HerA and NurA are required for DNA degradation. (A) A schematic illustrating nuclease activity analysis. (B) Analysis of DNA degradation of 3′-overhangs and blunt-ends in the presence and absence of ATP by a HerA-NurA mixture. HerA (27.8 nM hexamer) was mixed with NurA (83.4 nM dimer) and incubated at 65°C for 30 min. Samples were analyzed on a 10% native polyacrylamide gel. B, boiled samples. (C) Same as in (B), but the samples were analyzed on a 15% denaturing polyacrylamide, and only samples with ATP were analyzed. M, size marker. (D) A HerA-NurA complex containing a nuclease inactive form of NurA (D58A) loses DNA degradation activity. B, boiled samples. The concentrations of HerA and NurA(D58A) used for 3′-overhang substrates were 0.0625, 0.125, 0.25, 0.5, 1, and 2 μM. The concentrations of HerA and NurA(D58A) used for blunt-ended DNA substrates were 0.125, 0.25, 0.5, and 1 μM. [file 12867_2015_30_MOESM7_ESM.tiff]

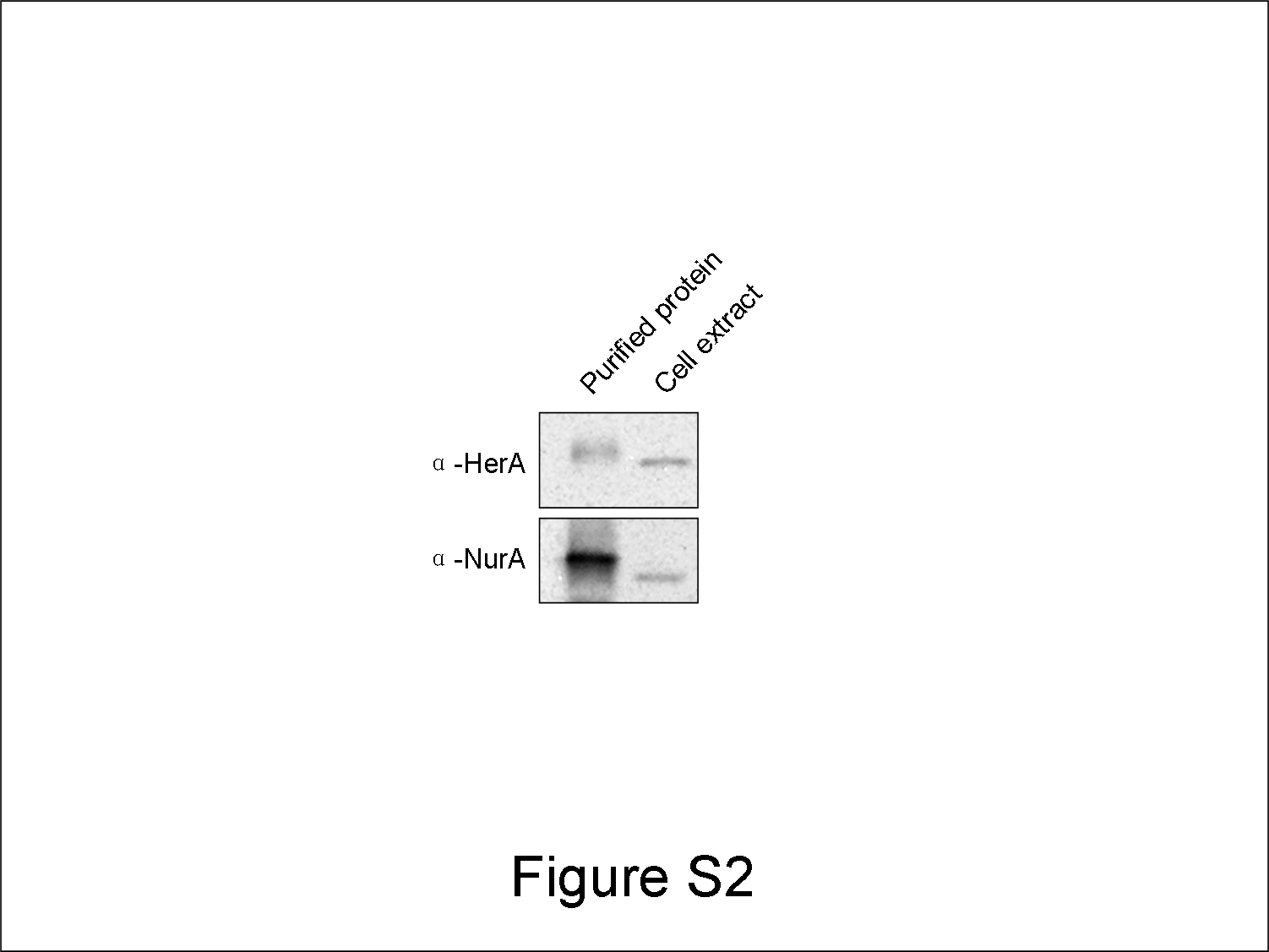

Supplement: Additional file 8: Figure S2. — Determination of HerA and NurA amounts in S. islandicus cells. Total proteins of wild type cells were separated by SDS-PAGE for quantitative Western blot. Purified HerA (19 ng) and NurA (45 ng) proteins from E. coli were loaded as standards for quantification. [file 12867_2015_30_MOESM8_ESM.tiff]
